# A qualitative investigation of the relevance of skin irritation and self-confidence bolt-ons and their conceptual overlap with the EQ-5D in patients with psoriasis

**DOI:** 10.1007/s11136-022-03141-y

**Published:** 2022-04-26

**Authors:** Fanni Rencz, Clara Mukuria, Alex Bató, Adrienn Katalin Poór, Aureliano Paolo Finch

**Affiliations:** 1 Department of Health Economics, Corvinus University of Budapest, 8 Fővám tér, 1093 Budapest, Hungary; 2 School of Health and Related Research (ScHARR), University of Sheffield, Sheffield, UK; 3 Károly Rácz Doctoral School of Clinical Medicine, Semmelweis University, Budapest, Hungary; 4 Department of Dermatology, Dermatooncology and Venereology, Semmelweis University, Budapest, Hungary; 5 EuroQol Office, EuroQol Research Foundation, Rotterdam, The Netherlands

**Keywords:** EQ-5D, Bolt-on, Psoriasis, EQ-PSO, Qualitative research

## Abstract

**Objectives:**

A number of bolt-ons have been proposed for the EQ-5D, including two psoriasis-specific bolt-ons, skin irritation and self-confidence. The study investigates and compares the relevance and comprehensiveness of these psoriasis-specific bolt-ons and the EQ-5D-5L and explores the potential conceptual overlaps between the existing five dimensions and the two bolt-ons.

**Methods:**

Psoriasis patients were purposively sampled according to age and gender. Semi-structured interviews, where participants were asked to complete the EQ-5D-5L and the bolt-ons while thinking aloud, were conducted. Probes were used to investigate the thought processes of patients regarding the dimensions, wording, recall period and relevant concepts not captured by the EQ-5D-5L and bolt-ons. Data were analysed thematically. A focus group was used to confirm the findings.

**Results:**

Overall, 16 patients completed the interviews. Sixteen and fifteen patients considered skin irritation and self-confidence relevant areas to describe psoriasis problems. Three patients considered itching a form of discomfort, and thus, pointed out a potential overlap between pain/discomfort and skin irritation. Twelve patients reported overall 10 general health- or psoriasis-related concepts that are not captured by the EQ-5D-5L, including itching, social relationships and sex life. Eleven patients reported that the recall period of the EQ-5D-5L might be subject to bias because of the daily or within-day fluctuations of their symptoms.

**Conclusions:**

The skin irritation and self-confidence bolt-ons contribute to improve content validity of the EQ-5D-5L in patients with psoriasis. The qualitative approach taken in this study expands the existing methodological framework for the development and testing validity of bolt-ons for the EQ-5D.

**Supplementary Information:**

The online version contains supplementary material available at 10.1007/s11136-022-03141-y.

## Introduction

Decision makers in healthcare rely on evidence from cost-utility analyses to judge the value of health interventions. Such analyses require health-related quality of life (HRQoL) data that enable the estimation of quality-adjusted life years (QALYs). The EQ-5D is the most commonly used HRQoL measure in estimating QALYs, recommended by over 20 health technology assessment bodies worldwide [1–3]. In general, the EQ-5D exhibits good validity and responsiveness across a broad range of health conditions and settings [4, 5]. However, there may be areas of HRQoL the general public or patients perceive as missing from the EQ-5D, limiting its content validity [6, 7]. Adding bolt-on dimensions to the EQ-5D specific to a certain patient population or culture is considered a solution to improve content validity. A recent systematic review identified 26 different bolt-on dimensions to the EQ-5D [8]. Examples include cognition [9], sleep [10], social relationships [11], vision, hearing and energy/tiredness [12]. Studies have used different methods to identify new bolt-on dimensions for EQ-5D including qualitative (e.g. interviews, focus group), quantitative (e.g. factor analysis, latent class analysis or pairwise choices) or mixed methods. [8, 11, 13, 14]. However, qualitative assessment of selected bolt-on dimensions is limited [15, 16].

The EQ-5D is one of the most frequently used generic measure of HRQoL in patients with psoriasis, including large observational studies and clinical trials [17–19]. Two psoriasis-specific bolt-ons (EQ-PSO) have been developed for the five-level EQ-5D (EQ-5D-5L), one physical (i.e. skin irritation) and one mental (i.e. self-confidence) [15]. Being among the first few bolt-ons, the EQ-PSO was primarily designed to explore methodological feasibility for the development of bolt-ons [15]. During the development of EQ-PSO, four candidate dimensions (skin irritation, skin appearance, self-confidence and social/relationship difficulties) were identified through a literature review, qualitative semi-structured interviews with patients and consultation with a clinical expert, and exploratory factor analysis was used to select the final two dimensions. Improved responsiveness of the EQ-PSO was confirmed in pooled data of three clinical trials using multiple language versions [20]. Although translations exist (n = 48), qualitative evidence documenting the content validity of the EQ-PSO in different languages and cultural contexts is only available from the development study undertaken in the UK [15]. Moreover, it is unclear if there are any conceptual overlaps between the skin irritation and self-confidence dimensions and the existing five dimensions. An earlier study with burn patients found a moderate correlation between itching as rated on a visual analogue scale and the pain/discomfort dimension of the EQ-5D-5L [21]. It may also be assumed that self-confidence is, to some extent, captured by the dimension of anxiety/depression.

The objectives of our study were therefore to (1) investigate and compare the relevance of the content, comprehensiveness and comprehensibility of EQ-5D-5L and EQ-PSO in Hungarian patients with psoriasis; and (2) explore the potential conceptual overlaps between the existing five dimensions and the two bolt-ons.

## Methods

The study design, data analysis and reporting followed the Consolidated criteria for reporting qualitative studies (COREQ) checklist [22].

### Study design and participants

Qualitative one-on-one interviews and a focus group were carried out between November 2020 and June 2021 among Hungarian psoriasis patients. Patients were recruited via a local psoriasis patient association in Budapest, Hungary. Patients were sampled using a convenience sampling technique. While stringent quotas were not used, the recruitment intended to achieve heterogeneity in the patient population in terms of age, gender and clinical characteristics, e.g. disease severity and types of treatment. Interviews were conducted until data saturation, whereby additional interviews did not generate any important new themes in the qualitative analysis [23]. Study inclusion criteria were as follows: i) aged 18 years of above; ii) diagnosed with psoriasis by a dermatologist; iii) cognitive ability to read and interpret questions in Hungarian and iv) signed written informed consent. A subgroup of the interviewed patients was invited to a focus group to discuss the findings of the thematic analysis. In response to the COVID-19 pandemic, online video interviewing was offered for those patients that refused the face-to-face interaction. Face-to-face interviews and the focus group were held in an office at the Corvinus University of Budapest. Patients received a gift card of 5,000 HUF (≈14 euro) in return for participation.

### Interviews

All interviews were conducted by two of the authors (F.R. and A.B.), both of whom had previous interviewing experience. The interview procedure combined elements of concurrent think aloud, where participants verbalize their thoughts while completing the questionnaire, and verbal probing [24]. We used this approach as we were interested in exploring patients’ underlying thought processes rather than exact responses to the questionnaire items. [25]. A topic guide was developed by the research team that included a dermatologist with experience in treating psoriasis patients. After the pilot-test with two patients, small revisions were made to the topic guide including adding a ranking question about the most and least relevant dimensions. The final interview guide comprised of three sections. In the first section, the aims of the study and the interview process were explained to each patient. Then they were asked about their experiences with psoriasis and important aspects of health and health-related quality of life and how these were impacted by psoriasis. In the second section, patients completed the EQ-5D-5L descriptive system, followed by the EQ VAS and EQ-PSO descriptive system (i.e. five core dimensions and the two bolt-ons). The ordering of the three measures was fixed across the interviews. Patients were asked to concurrently verbalise their thoughts when completing the questionnaires and probes were only used to further investigate these. In the last section, patients were asked to directly compare the EQ-5D-5L and EQ-PSO descriptive systems. At the end of each interview, patients completed a short questionnaire about their sociodemographic and clinical background.

### Focus group

A focus group was conducted to confirm the key results of the interviews and to provide an opportunity to clarify any uncertainties. Patients reporting an overlap between the bolt-ons and the five dimensions or providing useful suggestions on the improvement of bolt-ons were selectively invited to the focus group based on their responses during the individual interviews. The focus group was led by one of the authors (F.R.) using a topic guide developed by the research team specifically for the focus group. Another researcher (A.B.) was also present as an observer to take notes. At the beginning of the group discussion, patients were asked to fill in the EQ-PSO (including EQ VAS).

### Measures

The EQ-5D-5L comprises a five-dimension descriptive system and a visual analogue scale (EQ VAS) with endpoints of 0 (worst health you can imagine) and 100 (best health you can imagine) [26]. Each of the five dimensions of the descriptive system (mobility, self-care, usual activities, pain/discomfort and anxiety/depression) have five response options: no problems, slight problems, moderate problems, severe problems and extreme problems/unable to [27]. The summary of the responses on the five dimensions give the descriptive profile that can be converted into a utility value (index score) based on societal preferences. The EQ-PSO attaches two additional dimensions to the EQ-5D-5L, skin irritation and self-confidence, both with five response options [15]. For skin irritation, itching is mentioned as a supporting example both in the dimension heading and response options (‘I have no itching’, ‘I have slight itching’, etc.). The official Hungarian versions were used for both the EQ-5D-5L and EQ-PSO.

### Data analysis

All interviews as well as the focus group were audio-recorded and transcribed verbatim. Thematic analysis was carried out by three researchers with expertise in health outcomes research [28]. First, all transcripts were read by one of the authors and the initial themes were inductively derived. Categories and sub-categories were developed to describe emerging themes. A customized extraction matrix was developed in Microsoft Excel 2016 and categories and sub-categories were reported, with their associated interview IDs. Disagreements were resolved through regular discussions within the team. Quotes were selected to support the main categories and subcategories.

### Ethics

The study protocol was approved by the Research Ethics Committee of the Corvinus University of Budapest (No. KRH/342/2020).

## Results

### Sample characteristics

A total of 21 patients with psoriasis were recruited, three of whom withdrew. Two participants did not show up, resulting in a final sample of 16 patients. For 15 of them, we conducted the interview face-to-face. The mean interview duration was 59 min (range 41–91 min). No important new themes emerged after the eleventh interview, confirming that data saturation was attained. The sample had a good spread of age groups, was balanced in terms of gender, was well educated and had heterogeneous clinical characteristics. Detailed characteristics of the participants are presented in Table 1.

**Table 1:** Characteristics of psoriasis patients (n = 16)

Variables	Median (range) or n (%)
Age (years)	54 (22–72)
*Gender*	
Female	9 (56%)
Male	7 (44%)
*Education*	
Primary	1 (6%)
Secondary	4 (25%)
College/university	11 (69%)
*Employment*	
Employed full-time	8 (50%)
Employed part-time	1 (6%)
Unemployed	1 (6%)
Retired	6 (38%)
Disease duration (years)	23 (3–48)
*Number of body regions affected*	
1–2	2 (13%)
3–4	6 (38%)
≥ 5	8 (50%)
*Body regions affected*	
Scalp	12 (75%)
Face	5 (31%)
Auditory canals	2 (13%)
Arms	7 (44%)
Hands	3 (19%)
Fingernails	6 (38%)
Chest	5 (31%)
Back	6 (38%)
Abdomen	4 (25%)
Buttocks	7 (44%)
Genitals	2 (13%)
Thighs	9 (56%)
Legs	5 (31%)
Feet	5 (31%)
Toenails	1 (6%)
Self-reported severity VAS (0–10)*	5.3 (1–10)
Comorbidities	13 (81%)
Anxiety	7 (44%)
Cardiovascular disease	4 (25%)
Chronic tinnitus	1 (6%)
Depression	2 (13%)
Diabetes	1 (6%)
Gastroesophageal reflux disease	1 (6%)
Liver cirrhosis	1 (6%)
Musculoskeletal disease**	6 (38%)
Psoriatic arthritis	5 (31%)
Thyroid disease	2 (13%)
*Current treatment*	
Topical therapy	11 (69%)
Combination of topical and photo- and/or systemic non-biological therapy	4 (25%)
Biological therapy	1 (7%)
EQ VAS (0–100)	70 (30–100)

Figures may not total 100% due to rounding

*VAS*  visual analogue scale

^*^Endpoints: not severe at all = 0, extremely severe = 10

^**^Other than psoriatic arthritis

### Impact of psoriasis on HRQoL

**Fig. 1:**
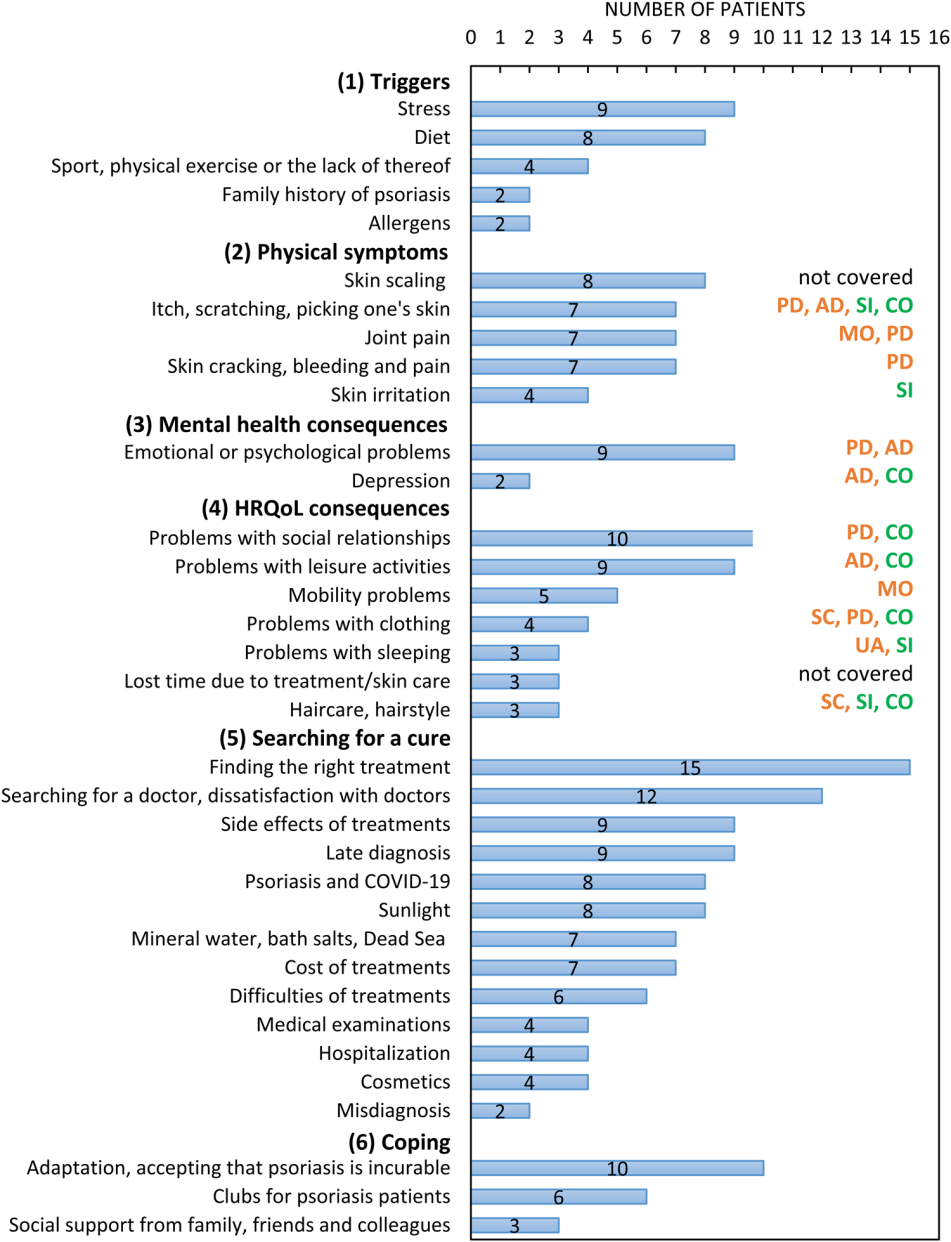
Concept map of the impact of psoriasis on patients’ lives and EQ-5D-5L and EQ-PSO content coverage. AD = anxiety/depression, CO = self-confidence, HRQoL = health-related quality of life, MO = mobility, PD = pain/discomfort, SC = self-care, SI = skin irritation, UA = usual activities. Orange dimensions refer to the EQ-5D-5L and green to the two psoriasis-specific bolt-ons

Patients reported 35 aspects on how psoriasis impacted on their lives which were grouped into six concepts: triggers, physical symptoms, mental health consequences, HRQoL consequences, searching for a cure and coping (Fig. 1). Skin scaling was the most commonly mentioned troubling symptom of psoriasis, followed by itching and scratching*.* A few patients experienced painful cracking and bleeding of the skin*.* Among adverse physical consequences of psoriasis, patients frequently reported problems with their social relationships, leisure activities, mobility and limitations in clothing choices due to their visible skin symptoms. These were connected to mental health consequences, such as feeling unattractive, being stared at by others and having to frequently inform others that psoriasis is not contagious or caused by poor hygiene.

### Comparing EQ-5D-5L and EQ-PSO

Results of the thematic analysis were summarized under the following themes: content of dimensions, overall relevance, missing concepts, ranking of dimensions, overlap of dimensions, suggested changes, response levels, EQ VAS and recall period.

### Content of the EQ-5D and EQ-PSO

Generally, patients interpreted the five dimensions as intended. However, three patients (19%) interpreted ‘dressing myself’ not as an ability to dress but rather as psoriasis influencing the clothes they can wear, e.g. *“My scalp is scaling so I cannot really wear black tops and this is very bad especially that I have to wear black at one of my workplaces and I continuously keep on shaking my top as it looks like I have dandruff”*. Another patient said that *“I also have a problem with washing myself because I have to pay attention to what cosmetics I use”*. There was a considerable agreement about the meaning of skin irritation since all patients responded to this dimension taking into account their perceived level of itch. However, four patients mentioned skin irritation being independent from itch and one patient emphasized the difference between these two: “*Well, it is also possible that a skin irritation hurts but does not itch. It is like all bugs are insects, but not all insects are bugs*”. For the majority of patients, self-confidence covered their problems with social relationships due to psoriasis and over one-third of patients interpreted this dimension as a belief in oneself or being a valued person.

### Relevance of the EQ-5D-5L and the EQ-PSO

The instructions in the descriptive systems were clear and easily understandable for all patients. In total 38% of the patients considered the EQ-5D-5L covered important aspects of HRQoL: *“Overall, I find the questionnaire good because this is what a human being is made of, and these are the most important ones [dimensions] in my opinion, too”*. Other patients expressed concerns about the questionnaire, such as irrelevance of some dimensions in the context of psoriasis: *“Well, I cannot connect these first four topics to psoriasis and myself”*. Two patients noticed the EQ-5D-5L is relevant for detecting the impact of severe psoriasis on patients and recognized the generic nature of the questionnaire. For example, *“It is much more relevant for those patients that have joint symptoms or whose condition is more severe”* and *“here, many of the questions are not related to psoriasis, but to the problems of an elderly person in general.”*

Sixteen (100%) and fifteen (94%) patients considered the skin irritation and self-confidence bolt-ons as important areas in psoriasis, respectively. All patients rated the EQ-PSO better than the EQ-5D-5L to describe their problems with health-related quality of life, e.g. “*the topics of the skin irritation and self-confidence which are, can be said, the two most important aspects of this problem or disease”.*

Figure 1 shows that the EQ-PSO was well aligned with nearly all important impacts of psoriasis raised by patients, with the exception of skin scaling and lost time due to having psoriasis.

### Missing concepts

Overall, 11 (69%) patients indicated 16 missing themes for the EQ-5D-5L and 12 (75%) patients indicated 11 missing themes for the EQ-PSO. These missing concepts were summarized under three large categories: general health-related, psoriasis-related and non-health-related concepts. In the EQ-5D-5L, the most commonly reported missing concepts were social relationships (*n* = 8) with a number of other concepts highlighted by three or less participants. Itching as a missing concept was raised by only one patient. Limitations in clothing (i.e. preference for covering visible skin symptoms or avoidance of wearing dark colours to hide flaking skin) was considered missing by two patients. There were fewer missing concepts identified in the EQ-PSO with social relationships identified by five participants (Table 2). The majority of the remaining themes were suggestions for other background information to solicit in a more general questionnaire designed for psoriasis patients, e.g. presence of psoriatic arthritis, activities given up since having been diagnosed with psoriasis or how patients’ eating choices affect their psoriasis.

**Table 2:** Missing concepts from the EQ-5D-5L and EQ-PSO

Concepts	EQ-5D-5L		EQ-PSO		Example quote
	*n*	%	*n*	%	
*GENERAL HEALTH-RELATED*					
Sex life	3	19	1	6	014: Certainly I would include how this affects, for instance, sex life
Stress and conflict management	1	6	1	6	006: In my opinion, [the questionnaire] did not ask about stress and conflict management
*PSORIASIS-RELATED*					
*Physical symptoms*					
Itching	1	6	0	0	010: These symptoms at the beginning, e.g. itching, there was horribly itchy and painful… maybe this is missing, too
Psoriatic arthritis	0	0	1	6	P001: I might still miss that it [psoriasis] extends to the joints and it would be possible to ask if it has already reached that stage at all or not
Skin oozing	0	0	1	6	013: Well, yes, this oozing, it is not listed here
*Assessment of psoriasis by others*					
Social relationships	8	50	5	31	004: [Psoriasis] makes dating harder anyway, let's say, I go to a party, I tie my hair up and when someone notices it, then obviously their first thought is not 'wow how nice is that', so yes, people cannot really deal with this for the first time
Clothing	2	13	0	0	010: In summer I put on the my thin knee-length pants made of canvas or a knee-length skirt no matter that I am full of patches and I do not care, but others may be bothered even by this
Hairstyle	2	13	0	0	001: It is a continuously recurring thing for me to try fixing my hair that it [my psoriasis] should not be visible
*Searching for a cure*					
Finding the right treatment	2	13	5	31	004: Perhaps I might add which lotions, solutions or medicines did you try up till now and which of these did you find effective?
What I do to feel better	1	6	2	13	002: What steps I have done to improve the already lost function or to maintain health that is the most important part in quality of life
Doctors' attitude	0	0	1	6	010: How doctors handle this thing [psoriasis], let's say, how much attention they pay to this
*Life changes*					
Changing workplace/profession	0	0	1	6	008: Perhaps one might have to change workplace/profession
Giving up leisure activities	2	13	1	6	010: [What is missing] if I dare to go to the swimming pool or how do I look like there?
Dietary awareness	3	19	3	19	001: I would ask who and to what extent is consciousness about nutrition
*NON-HEALTH-RELATED*					
Education and being connected to the arts	1	6	0	0	005: The education level or connectedness to the arts, to what extent a person's way of life is influenced by these, for example, theatre, a book or cinema, or it may even be a sport event
Financial situation	1	6	0	0	009: I would say 'background and financial situation' as a headline… marital status or housing conditions also belong here
Family situation	1	6	0	0	
Housing conditions	1	6	0	0	
Workplace	1	6	0	0	009: What has a great impact on quality of life is what one works
Travelling	1	6	0	0	001: …in addition to the things which to be done compulsory, it also includes things that improve quality of life very much, for example, leisure… not what I have to do or I am anxious about it or not because I do it. My quality of life is good because I go on a summer vacation once a year or go skiing so these really-really improve my quality of life

### Ranking of dimensions

In the EQ-5D-5L, over one-third of patients considered usual activities and anxiety/depression the most relevant dimensions, whereas half of the patients indicated self-care as being the least relevant one (Table 3). The relevance of dimensions substantially changed when the two bolt-ons were added to the EQ-5D-5L. In the EQ-PSO, skin irritation was identified as the most relevant dimension, followed by self-confidence, whereas self-care and anxiety/depression were the least relevant dimensions.

**Table 3:** Most and least relevant dimensions

Dimension*	EQ-5D-5L				EQ-PSO			
	Most relevant		Least relevant		Most relevant		Least relevant	
	*n*	%	*n*	%	*n*	%	*n*	%
Mobility	2	14	3	21	1	7	2	14
Self-care	0	0	7	50	1	7	4	29
Usual activities	5	36	1	7	0	0	3	21
Pain/discomfort	1	7	2	14	3	21	2	14
Anxiety/depression	5	36	2	14	2	14	4	29
Skin irritation	n/a	n/a	n/a	n/a	5	36	0	0
Self-confidence	n/a	n/a	n/a	n/a	4	29	1	7
All are relevant	1	7	0	0	1	7	0	0

^*^Only asked in 14 interviews and not in the pilot. One patient may have indicated none or multiple dimensions as most and least relevant. n/a = not applicable

### Overlap between dimensions

Several minor conceptual overlaps were identified between the seven dimensions (Table 4). Two patients described washing oneself as part of both self-care and usual activities, two patients reported a potential overlap between anxiety and discomfort and one patient reported an overlap between depression and discomfort.

**Table 4:** Overlapping dimensions

(Sub)dimension	Overlap	*n*	%	Example quote
Self-care (washing)	Usual activities	2	13	010: Washing myself is a usual activity, I don't have any problem with it
Anxiety	Discomfort	2	13	003: That is right, these [anxiety and discomfort] are the same since anxiety is a kind of discomfort when one cannot relax because of what the others may say…
Depression	Discomfort	1	6	005: The weather also affects the depression a bit, because when it is so ugly, foggy and gloomy weather this is so depressing for me
Skin irritation*	Pain	1	6	011: It is painful since it [my scalp] has been stretching
	Discomfort	3	19	004: I rather have a moderate discomfort, I do not have much pain in my joints, the patches do not hurt either, they rather itch
Self-confidence*	Anxiety	2	13	003: Anxiety could also belong to the self-confidence, because whoever does not have self-confidence they are probably anxious
	Depression	2	13	009: Self-confidence overlaps a bit with depression. It might be possible to rephrase 'depression' to ask if it causes lack of self-confidence

^*^ In total, three patients reported an overlap between skin irritation and pain/discomfort and three patients between self-confidence and anxiety/depression

For the two bolt-on dimensions, three patients considered ‘itch’ a form of discomfort, and thus, pointed out a potential overlap between pain/discomfort and skin irritation: *“I rather have a moderate discomfort, I do not have much pain in my joints, the patches do not hurt either, they rather itch”.* Self-confidence showed an overlap with anxiety/depression (anxiety *n* = 1, depression *n* = 1 and both *n* = 1) according to three patients.

### Suggested changes

Ten patients suggested changes in the EQ-5D-5L or EQ-PSO dimensions (Table 5). Changes included the use of wider descriptors for mobility so that it included other motor abilities and for skin irritation, by extending the range of symptoms described beyond itching (e.g. skin scaling, skin cracking or skin flaking). Few patients suggested adding further supporting examples to other dimensions, e.g. ‘flaking skin’ to usual activities, ‘frustration’ to pain/discomfort, ‘stress’ to anxiety/depression and ‘self-esteem’ to self-confidence. The self-care, pain/discomfort and anxiety/depression dimensions were sometimes suggested to be separated. Three wording changes were proposed, the replacement of ‘washing’ with ‘skin care’, ‘anxiety’ with ‘stress’ and ‘depression’ with ‘mood disorder’.

**Table 5:** Suggested changes in EQ-5D-5L and two bolt-on dimensions

Themes	*n*	%	Example quote
*Mobility*			
'Walking about' is too narrow, replace it by 'Moving (and sports)'	4	25	001: I would not limit mobility only to walking about; a healthy physique involves not only being able to walk about itself but other movements, such as if I can bend down, if it does not cramp or does not stretch anywhere
Need to define mobility in terms of both quantity and quality	1	6	006: The amount of time [you spend moving] or what kind of physical activity causes a limitation to you
*Self-care*			
Split it into 2 questions	1	6	012: Now what do I have a mild problem with, washing myself or getting dressed?… It should have been taken apart, at least
Replace 'washing yourself' by skincare	1	6	P002: For patients with psoriasis, another definition would be needed; suddenly 'skin care' comes to my mind which, of course, is equivalent to 'washing myself' but still is somehow different
*Usual activities*			
Add 'flaking skin'	1	6	013: When someone has many plaques, almost covered from head to toe, when they move, it [skin] falls down to the floor, you know, this silvery thing and after it should be vacuumed or swept
*Pain/discomfort*			
Split it into 2 questions	1	6	006: I don't know how I should answer that. I don't have any pain, but in the meantime, there is discomfort so I don't feel this is an appropriate response… I would separate it, yes, I would
Add 'frustration'	1	6	004: I would still add frustration here next to pain/discomfort
*Anxiety/depression*			
Split it into 2 questions	3	19	P002: Well, I would absolutely exclude depression, so I would not even put them [anxiety and depression] in the same [dimension]
Replace 'anxiety' with stress	1	6	P002: When I think of anxiety it reminds me something which makes me constantly anxious, some problem, something which is there all the time, but stress comes as if I may be stressed about something today but maybe I will be stressed again only on Saturday or Sunday… Well, I would not really call stress as anxiety… I would rather put 'stress' or 'everyday problems' here [instead of anxiety]
'Depression' is a too strong word, replace it with 'mood disorder'	2	13	002: Respondent: The word depression may be a bit too strong… Because of the stigma, so one knows that this is the problem but it is still quite bad to face this… *Interviewer: And what would you use instead?* Respondent: Let's say, mood disorder
Add 'stress'	1	6	006: I think stress would be more easy to understand for people, I might write stress next to depression
*Skin irritation*			
'Skin irritation' is too narrow	1	6	006: I see this [itching] as small as walking in mobility, the other topics are much bigger and more comprehensive… it is like, let's say, a little toe hurts or the middle and I take that out of pain, so I feel that this itch is just one thing in this whole problem…
Add 'skin scaling' and 'skin cracking'	3	19	004: I would add skin scaling and cracking to the irritation because, for me, when it is worse then it cracks
Add 'flaking skin'	1	6	P001: Psoriasis comes together with an excessive skin production and it is extremely horrible when one sweeps 200 g of skin from everywhere, everywhere… by the morning the skin gets dry and scaly, it is like snowing. Well, this may be added to skin irritation because it is a form [of skin irritation] too
Add ‘skin symptoms of bad smell’	1	6	002: I have a strong sense of smell… and in connection to skin irritation that it is not only irritation, for example, itch, but I specifically feel like my skin is rotting
*Self-confidence*			
Add 'self-esteem', 'self-identity', 'self-love'	1	6	006: Well, before self-confidence I would add self-esteem because this is a broad concept or I would still add self-identity and also self-love

### Response levels

Six patients (38%) reframed the response levels when completing the EQ-5D-5L or EQ-PSO (Online Resource 1). Of these, five patients used the response levels as a ‘frequency scale’ and one patient considered ‘level of bother’ for at least one dimension. This reframing most commonly occurred for mobility, pain/discomfort and skin irritation. Five patients reported problems with the level modifiers, including difficulty differentiating between levels 1 and 2 (for mobility) and levels 4 and 5 (for pain/discomfort, anxiety/depression and skin irritation).

### EQ VAS

Patients provided a wide range of interpretations of the endpoint labels ‘the best health you can imagine’ (= 100) and ‘the worst health you can imagine’ (= 0) (Online Resource 2). Most patients interpreted EQ VAS as generic and only a few patients used it as a psoriasis-specific scale. For example, when interpreting ‘0’ two patients referred to *“when one's whole body is covered with psoriasis”*. The instructions of EQ VAS were clear for the majority of patients; however, two patients mixed up the endpoints ‘0’ and ‘100’.

### Recall period

Several participants did not use the stated recall period but intended it as in general for the EQ-5D-5L (62%), EQ VAS (25%) and EQ-PSO (31%). Six participants reported that they would have provided identical answers if a different recall period had been asked: *“There is no difference between today and the other days of the week or other days of the month”*. Eleven patients reported that the recall period of the EQ-5D-5L and EQ-PSO might be subject to bias because of the daily or within-day fluctuations of their symptoms: “*in fact this is changing as dynamically if we had done this an hour ago I would have answered certain questions differently than now”*.

### Focus group

Eight patients were invited to the focus group, five of which attended (three women and two men). The discussions lasted 94 min (excluding the introduction of each patient). Three of the five patients considered itch a form of discomfort and responded to the EQ-PSO accordingly, whereas two patients reported their level of itching only in the skin irritation dimension. Of note, during the interviews these three patients did not mention itching as discomfort. All patients agreed that skin irritation is a broader category than itching that consists of other symptoms, such as skin scaling and plaquing. Although patients welcomed the idea to add these two symptoms as supportive examples to the skin irritation dimension, two patients cautioned against creating a double-barreled question and reported that their levels of itch and scaling are often not identical (e.g. slight itch and moderate scaling). Two patients described ‘skin cracking’ as a form of pain/discomfort, while two other patients considered it belonging to skin irritation. One patient said she would report skin cracking both on pain/discomfort and skin irritation. Patients came to a consensus that although self-confidence and anxiety/depression are related constructs there is no overlap between these two dimensions. Of note, one patient in the focus group was among the three patients that had suggested an overlap between self-confidence and anxiety/depression in the individual interviews.

## Discussion

This study used qualitative methods to investigate the EQ-5D-5L and EQ-PSO relevance among Hungarian psoriasis patients, focusing on potential conceptual overlap across dimensions. The results show that the EQ-5D-5L is considered relevant in psoriasis, but important concepts of HRQoL may still be missing from its descriptive system. Examples of them include general physical or mental health (e.g. stress, sex life) and psoriasis-specific health (e.g. itching, social relationships, dietary awareness). These findings extend the existing literature on important aspects of health and HRQoL the EQ-5D does not adequately capture in specific populations [6, 7]. Furthermore, some of the missing concepts identified by psoriasis patients are covered by already existing EQ-5D bolt-on dimensions, such as social relationships or sexual activity [11, 29].

The patient sample almost unanimously confirmed the relevance of the skin irritation and self-confidence bolt-on dimensions to their experience with psoriasis. The majority of patients described these as the two most important aspects of HRQoL in psoriasis. In most cases patients interpreted the EQ-5D-5L or EQ-PSO dimensions as generic and not related to any specific condition. However, in a few instances, certain words or phrases were interpreted as being related to psoriasis. The most prominent example was seen at the self-care dimension, whereby one-fifth of patients interpreted ‘dressing myself’ not as an ability to dress but rather as psoriasis influencing the clothes they can wear. Similarly, few patients used the EQ VAS as a scale that measures the proportion of body surface area affected by psoriasis. We believe that mode of administration and the context of the study may be, at least in part, responsible for this effect. Patients were aware of participating in a psoriasis-related interview and this might have led them to overly focus on their skin problems when completing the questionnaires.

A conceptual overlap was identified between the pain/discomfort and skin irritation dimensions and some patients reported itching as relevant to both dimensions. Yet other patients thought differently and reported itch only in the skin irritation dimension. These disagreements may be attributable to the multifaceted nature of psoriasis. Given the large variability of skin and joint symptoms, including different clinical manifestations, localisations and symptoms, no two patients experience psoriasis the same way. For example, the focus group discussion highlighted that some patients experience itch, while others only report skin scaling, and for others joint symptoms are more bothersome than those of the skin. Based on our results, the skin irritation dimension seems to be a useful bolt-on in this population that might cause some (minor) overlap with pain/discomfort. Conversely, the focus group confirmed that the self-confidence bolt-on dimension is independent from the other dimensions of the EQ-5D-5L and represents a standalone value.

Although patients identified several limitations of the EQ-PSO, one should also consider that adding two condition-specific items to the EQ-5D-5L cannot be expected to reflect all the important facets of psoriasis. Scale development and valuation implications, such as keeping the number, length and wording of the new dimensions reasonable should also be taken into account. It is possible therefore that what patients consider missing or suggest to improve may confront the researchers’ aims and a judgement has to be made as to whether those limitations are sufficient to warrant changing or adding to the measure. For example, during the concept elicitation section of our study, the problems caused by skin scaling were raised by more patients than itching. In agreement with this, several patients proposed extension of this dimension by adding other frequent psoriasis skin symptoms beyond itching. However, by doing so, one may risk of creating a double-barreled question in the skin irritation dimension. Furthermore, when valuing health states, members of the general population may easily imagine itching, while imagining other skin symptoms, such as skin scaling that they most likely have never experienced, could be challenging.

Given the EQ-PSO has a value set developed in the UK [15], our findings are of direct relevance to cost-utility analyses and subsequent reimbursement decisions by providing supporting evidence on the usefulness of the two bolt-on dimensions in psoriasis. Health technology assessment bodies, such as the National Institute for Health and Care Excellence (NICE) [30] in the UK and the Canadian Agency for Drugs & Technologies in Health (CADTH), have already accepted the EQ-PSO in recent submissions for psoriasis treatments’ appraisals [31].

Some limitations of the study need to be mentioned. A convenience sampling was used and the majority of patients were related to a patient association. Self-selection bias may also be present as patients voluntarily applied to the study. It is possible, for example, that willingness to participate in such an interview or being a member of a patient association are not independent from one’s self-reported health or self-confidence. Data were collected through two different modes of administration (face-to-face and video-interviewing) which could be a source of potential influence on responses but majority used one mode and there were no differences in what was reported across the modes. Another limitation is that as a substantially large pool of experimental bolt-on dimensions are available for the EQ-5D, it could have been possible to include further bolt-ons in our study, especially those that one may anticipate to be relevant for psoriasis patients (e.g. social relationships, sleep). Lastly, there may be minor differences in semantics between the Hungarian and other language versions of the EQ-5D-5L and EQ-PSO which may prevent the generalizability of these findings to other countries.

In summary, the skin irritation and self-confidence bolt-on dimensions are particularly pertinent and contribute to improve content validity of the EQ-5D-5L in patients with psoriasis. There is only minor conceptual overlap between the pain/discomfort and skin irritation that does not seem to detract from the added value of the bolt-on item. The qualitative approach taken in this study expands the existing methodological framework for the development and testing validity of bolt-ons for the EQ-5D.

## Supplementary Information

Below is the link to the electronic supplementary material.

Supplementary file1 (DOCX 20 KB)

## Data Availability

All data of this study are available from the corresponding author upon reasonable request.

## Code availability

N/A.

## References

[1] Kennedy-Martin M, Slaap B, Herdman M, van Reenen M, Kennedy-Martin T, Greiner W (2020). Which multi-attribute utility instruments are recommended for use in cost-utility analysis? A review of national health technology assessment (HTA) guidelines. The European Journal of Health Economics.

[2] Rencz F, Gulácsi L, Drummond M, Golicki D, Prevolnik Rupel V, Simon J (2016). EQ-5D in Central and Eastern Europe: 2000–2015. Quality of Life Research.

[3] Wang A, Rand K, Yang Z, Brooks R, Busschbach J (2021). The remarkably frequent use of EQ-5D in non-economic research. European Journal of Health and Economy.

[4] Feng YS, Kohlmann T, Janssen MF, Buchholz I (2021). Psychometric properties of the EQ-5D-5L: A systematic review of the literature. Quality of Life Research.

[5] Finch AP, Brazier JE, Mukuria C (2018). What is the evidence for the performance of generic preference-based measures? A systematic overview of reviews. The European Journal of Health Economics.

[6] Shah KK, Mulhern B, Longworth L, Janssen MF (2017). Views of the UK general public on important aspects of health not captured by EQ-5D. Patient.

[7] Efthymiadou O, Mossman J, Kanavos P (2019). Health related quality of life aspects not captured by EQ-5D-5L: Results from an international survey of patients. Health Policy.

[8] Geraerds AJLM, Bonsel GJ, Janssen MF, Finch AP, Polinder S, Haagsma JA (2021). Methods used to identify, test, and assess impact on preferences of bolt-ons: A systematic review. Value Health.

[9] Krabbe PF, Stouthard ME, Essink-Bot ML, Bonsel GJ (1999). The effect of adding a cognitive dimension to the EuroQol multiattribute health-status classification system. Journal of Clinical Epidemiology.

[10] Yang Y, Brazier J, Tsuchiya A (2014). Effect of adding a sleep dimension to the EQ-5D descriptive system: A "bolt-on" experiment. Medical Decision Making.

[11] Finch AP, Brazier JE, Mukuria C, Bjorner JB (2017). An exploratory study on using principal-component analysis and confirmatory factor analysis to identify bolt-on dimensions: The EQ-5D case study. Value Health.

[12] Yang Y, Rowen D, Brazier J, Tsuchiya A, Young T, Longworth L (2015). An exploratory study to test the impact on three "bolt-on" items to the EQ-5D. Value Health.

[13] Finch AP, Brazier J, Mukuria C (2021). Selecting bolt-on dimensions for the EQ-5D: Testing the impact of hearing, sleep, cognition, energy, and relationships on preferences using pairwise choices. Medical Decision Making.

[14] Finch AP, Brazier JE, Mukuria C (2019). Selecting bolt-on dimensions for the EQ-5D: Examining their contribution to health-related quality of life. Value Health.

[15] Swinburn P, Lloyd A, Boye KS, Edson-Heredia E, Bowman L, Janssen B (2013). Development of a disease-specific version of the EQ-5D-5L for use in patients suffering from psoriasis: Lessons learned from a feasibility study in the UK. Value Health.

[16] Sampson C, Addo R, Haywood P, Herdman M, Janssen B, Mulhern B (2019). Development of EQ-5D-5L bolt-ons for cognition and vision. Value in Health.

[17] Yang Y, Brazier J, Longworth L (2015). EQ-5D in skin conditions: An assessment of validity and responsiveness. The European Journal of Health Economics.

[18] Poor AK, Rencz F, Brodszky V, Gulacsi L, Beretzky Z, Hidvegi B (2017). Measurement properties of the EQ-5D-5L compared to the EQ-5D-3L in psoriasis patients. Quality of Life Research.

[19] Yfantopoulos J, Chantzaras A, Kontodimas S (2017). Assessment of the psychometric properties of the EQ-5D-3L and EQ-5D-5L instruments in psoriasis. Archives of Dermatological Research.

[20] Pickard AS, Gooderham M, Hartz S, Nicolay C (2017). EQ-5D health utilities: Exploring ways to improve upon responsiveness in psoriasis. Journal of Medical Economics.

[21] Spronk I, Bonsel GJ, Polinder S, van Baar ME, Janssen MF, Haagsma JA (2020). Exploring the relation between the EQ-5D-5L pain/discomfort and pain and itching in a sample of burn patients. Health and Quality of Life Outcomes.

[22] Tong A, Sainsbury P, Craig J (2007). Consolidated criteria for reporting qualitative research (COREQ): A 32-item checklist for interviews and focus groups. International journal for quality in health care.

[23] Guest G, Bunce A, Johnson L (2006). How Many Interviews Are Enough?: An Experiment with Data Saturation and Variability. Field Methods.

[24] Collins D (2003). Pretesting survey instruments: An overview of cognitive methods. Quality of Life Research.

[25] Kuusela H, Paul P (2000). A comparison of concurrent and retrospective verbal protocol analysis. American Journal of Psychology.

[26] Brooks R (1996). EuroQol: The current state of play. Health Policy.

[27] Herdman M, Gudex C, Lloyd A, Janssen M, Kind P, Parkin D (2011). Development and preliminary testing of the new five-level version of EQ-5D (EQ-5D-5L). Quality of Life Research.

[28] Knafl K, Deatrick J, Gallo A, Holcombe G, Bakitas M, Dixon J (2007). The analysis and interpretation of cognitive interviews for instrument development. Research in Nursing & Health.

[29] Jelsma J, Maart S (2015). Should additional domains be added to the EQ-5D health-related quality of life instrument for community-based studies? An analytical descriptive study. Population Health Metrics.

[30] National Institute for Health and Care Excellence (NICE). (2016). Single Technology Appraisal Ixekizumab for treating moderate to severe plaque psoriasis [ID904] Committee Papers. Retrieved October 15, 2021 from https://www.nice.org.uk/guidance/ta442/documents/committee-papers-3

[31] CADTH Common Drug Reviews. (2018). Pharmacoeconomic Review Report: Guselkumab (Tremfya): (Janssen Inc.): Indication: For the treatment of adult patients with moderate-to-severe plaque psoriasis who are candidates for systemic therapy or phototherapy. Ottawa (ON): Canadian Agency for Drugs and Technologies in Health. Retrieved October 15, 2021 from https://www.cadth.ca/sites/default/files/cdr/pharmacoeconomic/SR0530_Tremfya_PE_Report.pdf 30457783

