# Supplementary material for: A qualitative investigation of the relevance of skin irritation and self-confidence bolt-ons and their conceptual overlap with the EQ-5D in patients with psoriasis
Source: Qual Life Res. 2022 Apr 26;31(10):3049–60. doi: 10.1007/s11136-022-03141-y (PMC9039271; doi:10.1007/s11136-022-03141-y)
Supplement: Supplementary file 1 — Supplementary file1 (DOCX 20 KB) [file 11136_2022_3141_MOESM1_ESM.docx]

**Supplementary Information**

**A qualitative investigation of the relevance of skin irritation and self-confidence bolt-ons and their conceptual overlap with the EQ-5D in patients with psoriasis**

Fanni Rencz, Clara Mukuria, Alex Bató, Adrienn Katalin Poór, Aureliano Paolo Finch

**Correspondence**:

Dr Fanni Rencz

Department of Health Economics
Corvinus University of Budapest
Address: 8 Fővám tér, H-1093 Budapest, Hungary
E-mail: fanni.rencz@uni-corvinus.hu

**Online Resource 1** Comments on response levels

**Online Resource 2** Content of the EQ VAS

**Online Resource 1 Comments on response levels**

| **Themes** | **Dimension** | **n** | **%** | **Example quote** |
| --- | --- | --- | --- | --- |
| ***REFRAMED THE RESPONSE LEVELS*** | | | | |
| Reframed as a frequency scale | MO | 3 | 19% | 010: Mostly I marked 'slight problems' because this replaces rare for me.  014: I think 'severe problems' means daily, nearly permanent. |
|  | PD | 3 | 19% | 010: Because it is very rare and that is rather mild. |
|  | SI | 2 | 13% | 006: I know the phases, when it is moderate and when severe. I take it as 'severe' when it itches more often. |
| Reframed as 'level of bother' | MO, SC, UA | 1 | 6% | 011: I felt from the questionnaire that the first part is about [activities] that you can or cannot do, you can or cannot get dressed ... but obviously we [psoriasis patients] can do it, so am I, but if it bothers me in any activity there is a lot more here, it bothers me very-very much in a lot, lot of activities. |
| ***REPORTED PROBLEMS WITH LEVEL MODIFIERS*** | | | | |
| Too difficult to differentiate between levels 1 and 2 | MO | 2 | 13% | P002: Here I can mark more than one, because it does not limit so much to say 'slight'. |
| Too difficult to differentiate between levels 4 and 5 | PD | 1 | 6% | 001: Concerning such subjective topics as discomfort… it is hard for me to make a difference [between levels 4 and 5] ... for me both answers are equal so they would have the same weight. |
|  | AD | 1 | 6% | 001: I am very anxious, I am extremely anxious, so where are the differences between the levels? I am severely depressed or extremely depressed, it would be hard for me to make any difference. |
|  | SI | 2 | 13% | 011: For me, between extreme and severe, it is really interesting, but severe comes last [instead of extreme]. |
| Mild depression is a clinical diagnosis | AD | 1 | 6% | 002: According to my clinical condition, I have a mild depressive episode and mixed anxiety-depressive disorder. |
| 'Moderate' or 'medium' [Hungarian-specific] | PD, AD | 1 | 6% | 001: It [the questionnaire] uses similar words everywhere and here earlier I wondered a little for a moment why it did not put it that way that 'I am medium-anxious' or 'I am medium-depressed' there is no such thing as being 'moderately depressed'? |
| ***SUGGESTED A CHANGE*** | | | | |
| Increase the number of response levels | MO | 1 | 6% | 010: It suddenly came to my mind that sometimes a sort of 10-point scale is used in certain situations, perhaps that might be better. |
| More information next to the response levels | PD | 1 | 6% | 004: I would explain it a bit in parentheses what slight, moderate and severe mean. |

AD = anxiety/depression, CO = self-confidence, MO = mobility, PD = pain/discomfort, SC = self-care, SI = skin irritation, UA = usual activities.

**Online Resource 2 Content of the EQ VAS**

| **Theme** | **n** | **%** | **Example quote** |
| --- | --- | --- | --- |
| ***THE BEST HEALTH YOU CAN IMAGINE*** | | | |
| **In general** |  |  |  |
| Lack of pain | 4 | 25% | 004: Yes, it would be 100 if I did not have any pain |
| Healthy lifestyle (e.g. sport, diet) | 3 | 19% | P002: One does sport regularly, eats properly and is happy. |
| Youth | 3 | 19% | 005: Well, the best imaginable health, you know, has many components, one of them is having a very good health, for that you have to be a bit younger |
| Lack of illness (in general) | 2 | 13% | 007: I would not have any problem not even age-related diseases |
| Free of symptoms/free of health complaints | 2 | 13% | 002: I am completely free of symptoms and I have already forgotten that I had an experience like that with a disease |
| Unachievable | 2 | 13% | 012: There is no man on earth who is fit as a fiddle who was born lucky and 100% virus-free, disease-free, there is nothing wrong with him, has never been sick… 80-85 let's agree, I think that's the normal health of an average person |
| Happiness | 1 | 6% | P002: The best, who is obviously young, does sport regularly, eats properly and is happy. |
| Health or well-being of my family | 1 | 6% | 009: My health today is 100… Since everything was fine, my child is in a good place, I talked with the other two [children] |
| No need for using healthcare | 1 | 6% | 013: One does not need to visit the doctor or hospital |
| No discomfort, harmony | 1 | 6% | 005: If someone feels good and is balanced that is a very good thing |
| Lack of anxiety | 1 | 6% | 006: I have nothing to be anxious about… |
| Physical health, vitality | 1 | 6% | 001: When one feels so well in one's skin, and feels vital, that the word 'health' does not even go through their mind. |
| Physical and mental health | 1 | 6% | 014: As long as someone is young and does not even know how good it is because they do not feel what it is like to always have pain somewhere. I don't know if mental health belongs here, because that is the other side |
| **In relation to psoriasis** |  |  |  |
| I have no skin symptoms | 3 | 19% | 004: It would be 100 if, on the one hand, I did not have any pain, I were free of symptoms, so my scalp were not flaking regardless of having it [psoriasis], it did itch in every hour and were not painful or cracking |
| I do not have psoriasis | 2 | 13% | 003: Not to have psoriasis, so to get rid of this problem |
| ***THE WORST HEALTH YOU CAN IMAGINE*** | | | |
| **In general** |  |  |  |
| Unable to take care oneself | 6 | 38% | 013: Complete dependency on others, when one is unconscious, they have to wear a diaper, have to be fed and washed |
| Paralyzed/disabled | 3 | 19% | 004: Well, when I am not even able to stand up… |
| Extreme pain | 3 | 19% | 001: When someone thinks 'shoot me in the head', because even existence means a problem, there is that level of pain… |
| Severe/deadly disease | 3 | 19% | P001: If someone has a deadly disease e.g. cancer or after having a stroke or heart attack |
| Dying | 2 | 13% | 003: Shall I say now if someone is dying? |
| Poor mental health | 2 | 13% | 009: It is an anxiety because of something ... I would rather say it for a mental state, there I could mark a 0. |
| Depression, panic disorder | 1 | 6% | 010: If I had a bad depression because of this, that would be the worst |
| Grief | 1 | 6% | 009: on the day of my father's funeral... that morning, yes, if I had to answer to something like this then for sure [would have marked 0] |
| Dead | 1 | 6% | 007: When someone is already in the coffin. |
| Worse than dead | 1 | 6% | P001: For example, to be physically paralyzed, not able to walk or anything, this is worse than dead, in my opinion. |
| **In relation to psoriasis** | | | |
| My whole body would be covered with psoriasis | 2 | 13% | 011: If one's whole body was covered [with psoriasis] |
